# Adaptive Maximum Correntropy Gaussian Filter Based on Variational Bayes

**DOI:** 10.3390/s18061960

**Published:** 2018-06-17

**Authors:** Guoqing Wang, Zhongxing Gao, Yonggang Zhang, Bin Ma

**Affiliations:** 1 College of Automation, Harbin Engineering University, Harbin 150001, China; wangguoqing2014@hrbeu.edu.cn (G.W.); zhangyg@hrbeu.edu.cn (Y.Z.); 2 Chinese Ship Research and Design Center, Wuhan 430064, China; masanshao@163.com

**Keywords:** Gaussian filter, maximum correntropy criterion, variational Bayes, Kalman filter

## Abstract

In this paper, we investigate the state estimation of systems with unknown covariance non-Gaussian measurement noise. A novel improved Gaussian filter (GF) is proposed, where the maximum correntropy criterion (MCC) is used to suppress the pollution of non-Gaussian measurement noise and its covariance is online estimated through the variational Bayes (VB) approximation. MCC and VB are integrated through the fixed-point iteration to modify the estimated measurement noise covariance. As a general framework, the proposed algorithm is applicable to both linear and nonlinear systems with different rules being used to calculate the Gaussian integrals. Experimental results show that the proposed algorithm has better estimation accuracy than related robust and adaptive algorithms through a target tracking simulation example and the field test of an INS/DVL integrated navigation system.

## 1. Introduction

As the benchmark work in state estimation problems, the linear recursive Kalman filter (KF) has been applied in various applications, such as information fusion, system control, integrated navigation, target tracking, and GPS solutions [1,2,3,4]. It is then extended to nonlinear systems through different ways to approximate the nonlinear functions or filtering distributions. Using the Taylor series to linearize the nonlinear functions, the popular extended Kalman filter (EKF) [5] is obtained. To further improve the estimation accuracy of EKF, several sigma points based nonlinear filters have been proposed in recent decades, such as unscented Kalman filter (UKF) using unscented transform [6], cubature Kalman filter (CKF) according to cubature rules [7], and divided difference Kalman filter (DDKF) adopting the polynomial approximations [8]. All these filters can be regarded as special cases of Gaussian filter (GF) [9,10,11], where the noise distribution is assumed to be Gaussian.

However, when the measurements are polluted by non-Gaussian noise, such as impulsive inference or outliers, GF will have worse estimation results and even break down [12,13]. Besides the computation extensive methods including particle filter [14,15], Gaussian sum filter [16], and multiple model filters [17], the robust filters, such as Huber’s KF (HKF, also known as M-estimation) [18,19,20] and $H_{\infty }$ filter [21], are also intended for the contaminated measurements. Although the $H_{\infty }$ filter can obtain guaranteed bounded estimation error, it does not perform well under Gaussian noise [22]. The Huber’s M-estimation is a combined $l_{1}$ and $l_{2}$ norm filter that can effectively suppress the non-Gaussian noise [18,19,20]. Recently, the information theoretical measure correntropy has been used to incorporate the non-Gaussian noise [23,24,25,26,27,28]. According to the maximum correntropy criterion (MCC), a new robust filter known as the maximum correntropy Kalman filter (MCKF) has been proposed in [24], and it is also extended to nonlinear systems using EKF [27] and UKF [25,26,28]. Simulation results show that an MCC based GF (MCGF) may obtain better estimation accuracy than M-estimation when choosing a proper kernel bandwidth [23,24,25,26]. Even so, both MCGF and M-estimation still require the information of nominal measurement noise covariance, which may be unknown or time varying in some applications. In this situation, the performance of MCGF will degrade as shown in our simulation examples.

Traditionally, the unknown noise statistic can be estimated by the adaptive filter, such as the Sage–Husa filter [29] and fading memory filter [30]. One drawback of these recursion adaptive filters is that the previously estimated statistic of the last time instant will influence current estimation, which is not suitable for the case measurement noise having frequently changing statistics [31]. The recently proposed variational Bayes (VB) based adaptive filter avoids this limitation by VB approximation, and VB based GFs (VBGFs) for both linear [32] and nonlinear systems [9,33] have been proposed.

In this paper, we proposed an adaptive MCGF based on the VB approximation, which is especially useful for estimating the system state from the measurements with unknown covariance non-Gaussian noise. Typical applications include low-cost INS/GPS integrated navigation systems [32] and maneuvering target tracking [33]. To overcome the limitation of MCGF under unknown time varying measurement noise covariance, the VB method is utilised to improve the adaptivity of MCGF, which is achieved through the fixed-point iteration framework. As will be demonstrated in our simulation results, our proposed method has better estimation accuracy than related algorithms. Furthermore, various filters can be obtained by using different ways to calculate the Gaussian integrals.

The rest of this paper is given as follows. In Section 2, after briefly introducing the concept of correntropy, we give the general MCGF algorithm. In Section 3, we explain the main idea of the VB method and the procedure of embedding it into MCGF to obtain our proposed adaptive MCGF. Section 4 gives the experimental results of a typical target tracking model and an INS/DVL navigation system comparing with several related algorithms. Conclusions are made in the final section.

## 2. Gaussian Filter Based on the Maximum Correntropy Criterion

### 2.1. Correntropy

As a kind of similarity measure, the correntropy of random variables *X* and *Y* is defined as [24,25,26]

(1) $$V\left(X,Y\right)=E\left[\kappa \left(X,Y\right)\right]=\int \int \kappa \left(x,y\right)dF_{X,Y}\left(x,y\right),$$

where E[·] denotes expectation, $F_{X,Y}\left(x,y\right)$ is the joint density function, and κ(x,y) represents the Mercer kernel. The most popular Gaussian kernel is given as the following:

(2) $$\kappa \left(x,y\right)=G_{\sigma }\left(e\right)=\exp\left(-\frac{e^{2}}{2\sigma^{2}}\right),$$

where e=x−y, and σ>0 is the kernel bandwidth.

Then, taking the Taylor series expansion on the Gaussian correntropy, we obtain that

(3) $$V\left(X,Y\right)=\sum_{n=0}^{\infty }\frac{{\left(-1\right)}^{n}}{2^{n}\sigma^{2n}n!}E\left[{\left(X-Y\right)}^{2n}\right].$$

Obviously, it contains all the even moments of X−Y weighted by the kernel bandwidth σ. It enables us to capture high order information when applying the correntropy in signal processing. In practice, we can use the sampling data to estimate the real correntropy since the joint density function is usually unavailable.

### 2.2. Maximum Correntropy Gaussian Filter

In this paper, we consider the following nonlinear system with additive noise

(4) $$\begin{array}{c} x_{i}=f_{i-1}\left(x_{i-1}\right)+w_{i-1},\\ z_{i}=h_{i}\left(x_{i}\right)+v_{i}, \end{array}$$

where $x_{i}\in R^{n}$ is the system state at time *i* and $z_{i}\in R^{m}$ denotes the measurement. $f_{i}\left(\cdot \right)$ and $h_{i}\left(\cdot \right)$ represent the known nonlinear functions. In standard GF, the process noise $w_{i}$ and the measurement noise $v_{i}$ are assumed to be zero mean Gaussian noise sequences with known covariance $Q_{i}$ and $R_{i}$. The initial state $x_{0}$ has known mean ${\bar{x}}_{0}$ and covariance $P_{0}$.

In both GF and MCGF, the one step estimation ${\hat{x}}_{i}^{-}$ and its estimation covariance $P_{i}^{-}$ are obtained through:

(5) $${\hat{x}}_{i}^{-}=\int f_{i-1}\left(x_{i-1}\right)N\left(x_{i-1}|{\hat{x}}_{i-1},P_{i-1}\right)dx_{i-1},$$

(6) $$P_{i}^{-}=\int \left(f_{i-1}\left(x_{i-1}\right)-{\hat{x}}_{i}^{-}\right){\left(f_{i-1}\left(x_{i-1}\right)-{\hat{x}}_{i}^{-}\right)}^{T}N\left(x_{i-1}|{\hat{x}}_{i-1},P_{i-1}\right)dx_{i-1}+Q_{i-1}.$$

To further improve the robustness of GF, MCC has been applied on the derivation of measurement update of MCGF. Consider the following regression model based on Equations (4)–(6) [25]:

(7) $$\left[\begin{array}{c} {\hat{x}}_{i}^{-}\\ z_{i} \end{array}\right]=\left[\begin{array}{c} x_{i}\\ h_{i}\left(x_{i}\right) \end{array}\right]+\left[\begin{array}{c} \delta x_{i}\\ v_{i} \end{array}\right]$$

where $\delta x_{i}={{\hat{x}}_{i}^{-}-x}_{i}$. The covariance of ${\left[\delta x_{i}\;\;v_{i}\right]}^{T}$ is

(8) $$\left[\begin{array}{cc} P_{i}^{-} & 0\\ 0 & R_{i} \end{array}\right]=\left[\begin{array}{cc} S_{p,i}S_{p,i}^{T} & 0\\ 0 & S_{r,i}S_{r,i}^{T} \end{array}\right]=S_{i}S_{i}^{T}.$$

Multiplying $S_{i}^{-1}$ on both sides of Equation (7), we obtain

(9) $$e_{i}=D_{i}-W_{i},$$

where $D_{i}=S_{i}^{-1}\left[\begin{array}{c} {\hat{x}}_{i}^{-}\\ z_{i} \end{array}\right]$ and $W_{i}=S_{i}^{-1}\left[\begin{array}{c} x_{i}\\ h_{i}\left(x_{i}\right) \end{array}\right]$.

Then, the optimal estimation ${\hat{x}}_{i}$ under the MCC can be obtained through the following optimization problem:

(10) $${\hat{x}}_{i}=\underset{x_{i}}{\arg\max}J_{L}=\underset{x_{i}}{\arg\max}\sum_{k=1}^{m+n}G_{\sigma }\left(e_{i}\left(k\right)\right),$$

where $e_{i}\left(k\right)$ is the *k*-th element of $e_{i}$.

Equation (10) can be solved by:

(11) $$\frac{\partial J_{L}}{\partial x_{i}}={\left(\frac{\partial h_{i}\left(x_{i}\right)}{\partial x_{i}}\right)}^{T}C_{i}\left(D_{i}-W_{i}\right)=0,$$

where

(12) $$C_{i}=\left[\begin{array}{cc} {\tilde{C}}_{x,i}, & 0,\\ 0, & {\tilde{C}}_{y,i}, \end{array}\right],$$

(13) $${\tilde{C}}_{x,i}=diag\left(G_{\sigma }\left(e_{i}\left(1\right)\right),\ldots ,G_{\sigma }\left(e_{i}\left(n\right)\right)\right),$$

(14) $${\tilde{C}}_{y,i}=diag\left(G_{\sigma }\left(e_{i}\left(n+1\right)\right),\ldots ,G_{\sigma }\left(e_{i}\left(n+m\right)\right)\right).$$

Note here that the diag(·) is used to denote the diagonal matrix.

Based on the above equation and using ${\hat{x}}_{i}^{-}$ to replace the $x_{i}$ contained in Equation (9), MCGF can be written in a similar way as GF except a modified measurement noise covariance [25]:

(15) $${\tilde{R}}_{i}=S_{r,i}{\tilde{C}}_{y,i}^{-1}S_{r,i}^{T}.$$

Then, ${\hat{x}}_{i}$ and its covariance $P_{i}$ can be obtained through

(16) $${\hat{x}}_{i}={\hat{x}}_{i}^{-}+K_{i}\left(z_{i}-{\hat{z}}_{i}\right),$$

(17) $$P_{i}=P_{i}^{-}-K_{i}P_{zz}K_{i}^{T},$$

where

(18) $$K_{i}=P_{xz}P_{zz}^{-1},$$

(19) $${\hat{z}}_{i}=\int h_{i}\left(x_{i}\right)N\left(x_{i}|{\hat{x}}_{i}^{-},P_{i}^{-}\right)dx_{i},$$

(20) $$P_{zz}=\int \left(h_{i}\left(x_{i}\right)-{\hat{z}}_{i}\right){\left(h_{i}\left(x_{i}\right)-{\hat{z}}_{i}\right)}^{T}N\left(x_{i}|{\hat{x}}_{i}^{-},P_{i}^{-}\right)dx_{i}+{\tilde{R}}_{i},$$

(21) $$P_{xz}=\int \left(x_{i}-{\hat{x}}_{i}^{-}\right){\left(h_{i}\left(x_{i}\right)-{\hat{z}}_{i}\right)}^{T}N\left(x_{i}|{\hat{x}}_{i}^{-},P_{i}^{-}\right)dx_{i}.$$

We easily find that the main difference between MCGF and GF is the modified measurement noise covariance, and MCGF shows excellent estimation performance when measurement is polluted by outliers or shot noise [24,25,26]. However, it still requires the knowledge of measurement noise covariance. When the covariance changes over time (which implies the true covariance is different from the known covariance), the MCGF algorithm does not perform well. Therefore, we adopt the adaptive method to further improve the performance of MCGF in this case.

## 3. Variation Beysian Maximum Correntropy Gaussian Filter

The main idea under state estimation is to obtain the posterior probability density function $p(x_{i}|z_{1:i})$. For GF, we obtain it through the Gaussian approximation $p\left(x_{i}|z_{1:i}\right)\approx N\left(x_{i}|{\hat{x}}_{i},P_{i}\right)$. However, if the measurement noise covariance $R_{i}$ is unavailable, we need to estimate the joint posterior distribution $p(x_{i},R_{i}|z_{1:i})$. This distribution can be solved by the free form VB approximation [9,32]:

(22) $$p\left(x_{i},R_{i}|z_{1:i}\right)\approx Q\left(x_{i}\right)Q\left(R_{i}\right),$$

where $Q(x_{i})$ and $Q(R_{i})$ are unknown approximation densities, which can be calculated by minimizing the Kullback–Leibler (KL) divergence between the true one and its corresponding approximation [9,32]:

(23) $$Q\left(x_{i}\right)\propto \exp\left(\int \log p\left(z_{i},x_{i},R_{i}|z_{1:i-1}\right)Q\left(R_{i}\right)dR_{i}\right),$$

(24) $$Q\left(R_{i}\right)\propto \exp\left(\int \log p\left(z_{i},x_{i},R_{i}|z_{1:i-1}\right)Q\left(x_{i}\right)dx_{i}\right).$$

According to the VB method, $p(x_{i},R_{i}|z_{1:i})$ can be approximated as a product of Gaussian distribution and inverse Wishart (IW) distribution [9,32]:

(25) $$p\left(x_{i},R_{i}|z_{1:i}\right)\approx N\left(x_{i}|{\hat{x}}_{i},P_{i}\right)\mathrm{IW}\left(R_{i}|v_{i},V_{i}\right),$$

where

(26) $$N\left(x_{i}|{\hat{x}}_{i},P_{i}\right)\propto |P_{i}{|}^{-1/2}\exp\left(-\frac{1}{2}{\left(x_{i}-{\hat{x}}_{i}\right)}^{T}P_{i}^{-1}\left(x_{i}-{\hat{x}}_{i}\right)\right)$$

(27) $$\mathrm{IW}\left(R_{i}|v_{i},V_{i}\right)\propto |R_{i}{|}^{-(v_{i}+n+1)/2}\exp\left(-\frac{1}{2}\mathrm{tr}\left(V_{i}R_{i}\right)\right),$$

where tr(·) is the trace of a matrix, and $v_{i}$ and $V_{i}$ are the degree of freedom parameter and the inverse scale matrix, respectively.

The integrals in (22) and (23) can be computed as follows [9,32]:

(28) $$\begin{array}{c} \int \log p\left(z_{i},x_{i},R_{i}|z_{1:i-1}\right)Q\left(R_{i}\right)dR_{i}\\ =-\frac{1}{2}{\left(z_{i}-h_{i}\left(x_{i}\right)\right)}^{T}{\langle R_{i}^{-1}\rangle }_{R}\left(z_{i}-h_{i}\left(x_{i}\right)\right)-\frac{1}{2}{\left(x_{i}-{\hat{x}}_{i}^{-}\right)}^{T}{\left(P_{i}^{-}\right)}^{-1}\left(x_{i}-{\hat{x}}_{i}^{-}\right)+C_{1}, \end{array}$$

(29) $$\begin{array}{c} \int \log p\left(z_{i},x_{i},R_{i}|z_{1:i-1}\right)Q\left(x_{i}\right)dx_{i}\\ =-\frac{1}{2}\left(v_{i}^{-}+n+2\right)\log|R_{i}|-\frac{1}{2}\mathrm{tr}\left(V_{i}^{-}R_{i}^{-1}\right)-\frac{1}{2}{\langle {\left(z_{i}-h_{i}\left(x_{i}\right)\right)}^{T}R_{i}^{-1}\left(z_{i}-h_{i}\left(x_{i}\right)\right)\rangle }_{x}+C_{2}, \end{array}$$

where ${\langle \cdot \rangle }_{R}=\int \left(\cdot \right)Q\left(R_{i}\right)dR_{i}$, ${\langle \cdot \rangle }_{x}=\int \left(\cdot \right)Q\left(x_{i}\right)dx_{i}$, and $C_{1}$, $C_{2}$ are some constants. Due to the fact that $Q\left(R_{i}\right)=\mathrm{IW}\left(R_{i}|v_{i},V_{i}\right)$, we obtain

(30) $${\langle R_{i}^{-1}\rangle }_{R}=\left(v_{i}-n-1\right)V_{i}^{-1}.$$

Besides this, the expectation can be rewritten as

(31) $${\langle {\left(z_{i}-h_{i}\left(x_{i}\right)\right)}^{T}R_{i}^{-1}\left(z_{i}-h_{i}\left(x_{i}\right)\right)\rangle }_{x}=\mathrm{tr}\left\{{\langle {\left(z_{i}-h_{i}\left(x_{i}\right)\right)}^{T}\left(z_{i}-h_{i}\left(x_{i}\right)\right)\rangle }_{x}R_{i}^{-1}\right\}.$$

Substituting (30) and (31) into (28) and (29), and matching the parameters in (26) and (27), we can obtain the following results:

(32) $$\begin{array}{c} v_{i}=v_{i}^{-}+1, \end{array}$$

(33) $$\begin{array}{c} T_{i}=\int \left(h_{i}\left(x_{i}\right)-{\hat{z}}_{i}\right){\left(h_{i}\left(x_{i}\right)-{\hat{z}}_{i}\right)}^{T}N\left(x_{i}|{\hat{x}}_{i}^{-},P_{i}^{-}\right)dx_{i}+{\left(v_{i}-n-1\right)}^{-1}V_{i}, \end{array}$$

(34) $$\begin{array}{c} K_{i}=P_{xz}{\left(T_{i}\right)}^{-1}, \end{array}$$

(35) $$\begin{array}{c} {\hat{x}}_{i}={\hat{x}}_{i}^{-}+K_{i}\left(z_{i}-{\hat{z}}_{i}\right), \end{array}$$

(36) $$\begin{array}{c} P_{i}=P_{i}^{-}-K_{i}T_{i}{K_{i}}^{T}, \end{array}$$

(37) $$\begin{array}{c} V_{i}=V_{i}^{-}+\int \left(z_{i}-h_{i}\left(x_{i}\right)\right){\left(z_{i}-h_{i}\left(x_{i}\right)\right)}^{T}N\left(x_{i}|{\hat{x}}_{i},P_{i}\right)dx_{i}, \end{array}$$

where ${\left(v_{i}-n-1\right)}^{-1}V_{i}$ is the estimated measurement covariance.

The VB based GF works well for unknown measurement noise covariance. However, when the measurement contains outliers or shot noise, their estimation will degrade, as will be shown in our simulation results. To overcome the shortcomings of MCGF and VBGF, we take the advantages of VB and MCC by the fixed-point iteration method, and design the so called VBMCGF algorithm, which is summarized as follows:

Step 1: **Predict:**
  ${\hat{x}}_{i}^{-}$ and $P_{i}^{-}$ are obtained through (5) and (6), and
  (38) $$v_{k}^{-}=\rho \left(v_{k-1}-n-1\right)+n+1,$$
  (39) $$V_{k}^{-}=BV_{k-1}B^{T},$$
  where 0<ρ≤1, 0<∣B∣≤1, and a reasonable choice is $B=\sqrt{\rho }I$.
Step 2: **Update:**
  First, set ${\hat{x}}_{i}^{\left(1\right)}={\hat{x}}_{i}^{-}$, $P_{i}^{\left(1\right)}=P_{i}^{-}$, $v_{i}=1+v_{i}^{-}$, and $V_{i}^{\left(1\right)}=V_{i}^{-}$. Calculate ${\hat{z}}_{i}$ and $P_{xz}$ by (19) and (21).
  For j=1,…,N, iterate the following equations:
  (40) $$\begin{array}{c} R_{i}^{\left(j\right)}=S_{r,i}^{\left(j\right)}{\left(S_{r,i}^{\left(j\right)}\right)}^{T}={\left(v_{i}-n-1\right)}^{-1}V_{i}^{\left(j\right)}, \end{array}$$
  (41) $$\begin{array}{c} e_{r,i}^{\left(j\right)}={\left(S_{r,i}^{\left(j\right)}\right)}^{-1}\left(z_{i}-{\hat{z}}_{i}\right), \end{array}$$
  (42) $$\begin{array}{c} {\tilde{C}}_{r,i}^{\left(j\right)}=diag\left(G_{\sigma }\left(e_{r,i}^{\left(j\right)}\left(1\right)\right),\ldots ,G_{\sigma }\left(e_{r,i}^{\left(j\right)}\left(m\right)\right)\right), \end{array}$$
  (43) $$\begin{array}{c} {\tilde{R}}_{i}^{\left(j\right)}=S_{r,i}^{\left(j\right)}{\left({\tilde{C}}_{r,i}^{\left(j\right)}\right)}^{-1}{\left(S_{r,i}^{\left(j\right)}\right)}^{T}, \end{array}$$
  (44) $$\begin{array}{c} T_{i}^{\left(j+1\right)}=\int \left(h_{i}\left(x_{i}\right)-{\hat{z}}_{i}\right){\left(h_{i}\left(x_{i}\right)-{\hat{z}}_{i}\right)}^{T}N\left(x_{i}|{\hat{x}}_{i}^{-},P_{i}^{-}\right)dx_{i}+{\tilde{R}}_{i}^{\left(j\right)}, \end{array}$$
  (45) $$\begin{array}{c} K_{i}^{\left(j+1\right)}=P_{xz}{\left(T_{i}^{\left(j+1\right)}\right)}^{-1}, \end{array}$$
  (46) $$\begin{array}{c} {\hat{x}}_{i}^{\left(j+1\right)}={\hat{x}}_{i}^{-}+K_{i}^{\left(j+1\right)}\left(z_{i}-{\hat{z}}_{i}\right), \end{array}$$
  (47) $$\begin{array}{c} P_{i}^{\left(j+1\right)}=P_{i}^{-}-K_{i}^{\left(j+1\right)}T_{i}^{\left(j+1\right)}{\left(K_{i}^{\left(j+1\right)}\right)}^{T}, \end{array}$$
  (48) $$\begin{array}{c} V_{i}^{\left(j+1\right)}=V_{i}^{-}+\int \left(z_{i}-h_{i}\left(x_{i}\right)\right){\left(z_{i}-h_{i}\left(x_{i}\right)\right)}^{T}N\left(x_{i}|{\hat{x}}_{i}^{\left(j+1\right)},P_{i}^{\left(j+1\right)}\right)dx_{i}, \end{array}$$
  End For. In addition, set $V_{i}=V_{i}^{\left(N+1\right)}$, ${\hat{x}}_{i}={\hat{x}}_{i}^{\left(N+1\right)}$, and $P_{i}=P_{i}^{\left(N+1\right)}$.

The main difference between the proposed VBMCGF and existing GFs lies in the modified estimation error covariance ${\tilde{R}}_{i}^{\left(j\right)}$, where VB iterations are used to estimate its value and MCC is used to modify it in the presence of non-Gaussian noises. The kernel bandwidth σ plays an important role in reducing the effect of non-Gaussian noise or outliers. A smaller σ will make the filter more sensitive to outliers, but it may affect the convergence performance. In addition, a too large σ may cause the VBMCGF to perform more like VBGF (It can be proved that, if σ→∞, the proposed VBMCGF will reduce to VBGF). One possible way to select it is by the trial and error method [24,25,26]. Another important issue is the number of fixed-point iterations. In fact, only a few iterations (e.g., 2 or 3) are enough [31,32].

As the general framework, our filter can be easily implemented according to the real requirements. For linear systems that are described by $x_{i}=F_{i-1}x_{i-1}+w_{i-1}$ and $z_{i}=H_{i}x_{i}+v_{i}$, the predation update in the VBMCKF is the same as KF:

(49) $${\hat{x}}_{i}^{-}=F_{i-1}{\hat{x}}_{i-1},$$

(50) $$P_{i}^{-}=F_{i-1}P_{i-1}F_{i-1}^{T}+Q_{i-1}.$$

In addition, the ${\hat{z}}_{i}$, $P_{xz}$, $T_{i}^{\left(j+1\right)}$, and $V_{i}^{\left(j+1\right)}$ that appeared in VBMCKF will reduce to the following equations:

(51) $${\hat{z}}_{i}=H_{i}{\hat{x}}_{i}^{-},$$

(52) $$P_{xz}=P_{i}^{-}H_{i}^{T},$$

(53) $$T_{i}^{\left(j+1\right)}=H_{i}P_{i}^{-}H_{i}^{T}+{\tilde{R}}_{i}^{\left(j\right)},$$

(54) $$V_{i}^{\left(j+1\right)}=V_{i}^{-}+H_{i}P_{i}^{\left(j+1\right)}H_{i}^{T}+\left(z_{i}-H_{i}{\hat{x}}_{i}^{\left(j+1\right)}\right){\left(z_{i}-H_{i}{\hat{x}}_{i}^{\left(j+1\right)}\right)}^{T},$$

while other steps are the same as the general framework.

When it comes to the nonlinear systems, the Gaussian integrals contained in ${\hat{x}}_{i}^{-}$, $P_{i}^{-}$, ${\hat{z}}_{i}$, $P_{xz}$, $T_{i}^{\left(j+1\right)}$, and $V_{i}^{\left(j+1\right)}$ can be calculated according to Taylor series, unscented transform, or cubature rules, and the corresponding filters are called VBMCEKF, VBMCUKF, and VBMCCKF, respectively.

## 4. Experimental Results

### 4.1. Simulation Results of the Target Tracking Model

To illustrate the performance of the proposed algorithm, we first give the simulation results using a typical target tracking model, where cubature rules are used to calculate the integrals. We compare the estimation accuracy of seven filters: CKF [7], MCCKF-1 [26], MCCKF-2 [25], VBCKF [9], HCKF [18], VBHCKF (which adopts Huber’s function) and the proposed VBMCCKF under various kinds of measurement noise. The target tracking example is modeled as [2]:

(55) $$x_{i}=\left[\begin{array}{cccc} 1 & T & 0 & 0\\ 0 & 1 & 0 & 0\\ 0 & 0 & 1 & T\\ 0 & 0 & 0 & 1 \end{array}\right]x_{i-1}+\left[\begin{array}{cc} \frac{T^{2}}{2} & 0\\ T & 0\\ 0 & \frac{T^{2}}{2}\\ 0 & T \end{array}\right]w_{i-1},$$

(56) $$z_{i}=\left[\begin{array}{c} \sqrt{{\left(\xi_{x}-\xi_{0}\right)}^{2}+{\left(\eta_{y}-\eta_{0}\right)}^{2}}\\ \arctan\frac{\eta_{x}-\eta_{0}}{\xi_{y}-\xi_{0}} \end{array}\right]+v_{i},$$

where $x_{i}={\left[\begin{array}{cccc} \xi_{x,i} & {\dot{\xi }}_{x,i} & \eta_{y,i} & \dot{\eta_{y,i}} \end{array}\right]}^{T}$ is the system state with $\left(\xi_{x,i},\eta_{y,i}\right)$ and $\left({\dot{\xi }}_{x,i},{\dot{\eta }}_{y,i}\right)$ being the position and velocity in *x* and *y* directions. The position of radar is set as $\left(\xi_{0},\eta_{0}\right)$ = (−100 m, −100 m). We set the sampling period T=0.1s, $Q_{i}=\mathrm{diag}\left(\begin{array}{cc} 0.04\;m^{2}s^{-3}, & 0.04\;s^{-3} \end{array}\right)$. The initial state is ${\hat{x}}_{0}={\bar{x}}_{0}={\left[-40\;m\;\;3\;{\mathrm{ms}}^{-1}\;\;10\;m\;\;1\;{\mathrm{ms}}^{-1}\right]}^{T}$ and the covariance is $P_{0}=\mathrm{diag}\left(\begin{array}{cccc} 4\;m^{2}, & 0.01\;m^{2}s^{-2}, & 4\;m^{2}, & 0.01\;m^{2}s^{-2} \end{array}\right)$. The root mean square error (RMSE) and average RMSE (ARMSE) in position or velocity are used to describe the estimation accuracy—for example, the RMSE and ARMSE in position are defined as [2]:

(57) $${\mathrm{RMSE}}_{pos}\left(i\right)=\sqrt{\frac{1}{M}\sum_{c=1}^{M}\left({\left(\xi_{x,i}^{c}-{\hat{\xi }}_{x,i}^{c}\right)}^{2}+{\left(\eta_{y,i}^{c}-{\hat{\eta }}_{y,i}^{c}\right)}^{2}\right)},$$

(58) $${\mathrm{ARMSE}}_{pos}=\frac{1}{L}\sum_{i=1}^{L}{\mathrm{RMSE}}_{pos}\left(i\right),$$

where $\left(\xi_{x,i}^{c},\eta_{y,i}^{c}\right)$ and $\left({\hat{\xi }}_{x,i}^{c},{\hat{\eta }}_{y,i}^{c}\right)$ are the true and estimated position in the *c*th Monte Carlo experiment, respectively. The RMSE and ARMSE of velocity are similar.

We here consider the following five kinds of measurement noises:

**Case A**: Gaussian distribution

(59) $$v_{i}∼N\left(0,\mathrm{diag}\left[{\left(0.2\;m\right)}^{2},{\left(0.015\;\mathrm{rad}\right)}^{2}\right]\right),$$

**Case B**: Time varying measurement noise covariance

(60) $$v_{i}∼N\left(0,\alpha_{i}^{2}\mathrm{diag}\left[{\left(0.2\;m\right)}^{2},{\left(0.015\;\mathrm{rad}\right)}^{2}\right]\right),$$

**Case C**: Gaussian mixture noise with time varying measurement noise covariance

(61) $$v_{i}∼0.8N\left(0,\alpha_{i}^{2}\mathrm{diag}\left[{\left(0.2\;m\right)}^{2},{\left(0.015\;\mathrm{rad}\right)}^{2}\right]\right)+0.2N\left(0,\mathrm{diag}\left[{\left(5\;m\right)}^{2},{\left(0.75\;\mathrm{rad}\right)}^{2}\right]\right),$$

**Case D**: Time varying measurement noise covariance and shot noise

(62) $$v_{i}∼{\left[\beta_{i},\gamma_{i}\right]}^{T}+N\left(0,\alpha_{i}^{2}\mathrm{diag}\left[{\left(0.2\;m\right)}^{2},{\left(0.015\;\mathrm{rad}\right)}^{2}\right]\right),$$

**Figure 1:**
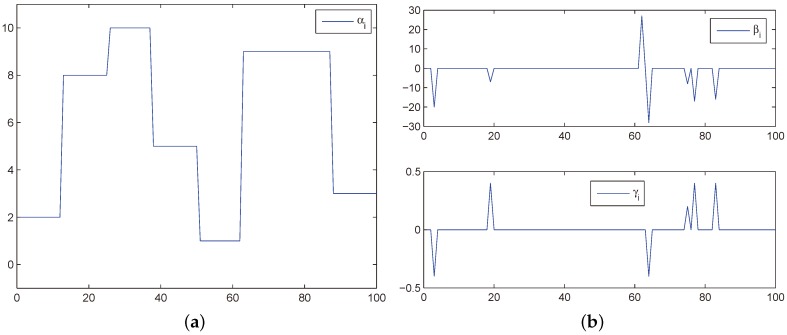
The time varying parameters. (**a**) $\alpha_{i}$; (**b**) $\beta_{i}$ and $\gamma_{i}$.

**Case E**: Gaussian mixture noise with time varying measurement noise covariance and shot noise

(63) $$v_{i}∼{\left[\beta_{i},\gamma_{i}\right]}^{T}+0.8N\left(0,\alpha_{i}^{2}\mathrm{diag}\left[{\left(0.2\;m\right)}^{2},{\left(0.015\;\mathrm{rad}\right)}^{2}\right]\right)+0.2N\left(0,\mathrm{diag}\left[{\left(5\;m\right)}^{2},{\left(0.75\;\mathrm{rad}\right)}^{2}\right]\right),$$

where the parameters $\alpha_{i}$, $\beta_{i}$, and $\gamma_{i}$ are given in Figure 1.

**Figure 2:**
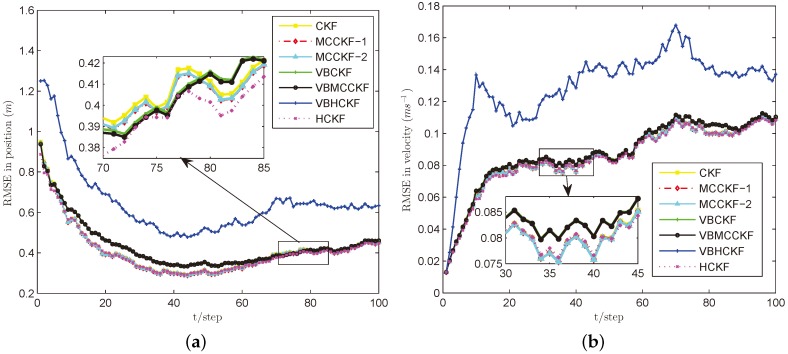
RMSE performances of different filters under Case A. (**a**) position; (**b**) velocity.

**Figure 3:**
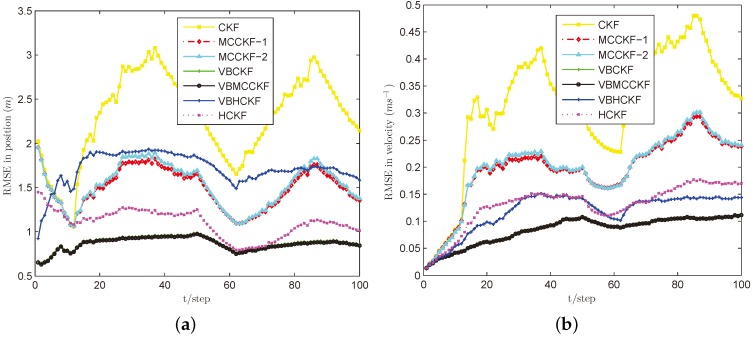
RMSE performances of different filters under Case B. (**a**) position; (**b**) velocity.

**Figure 4:**
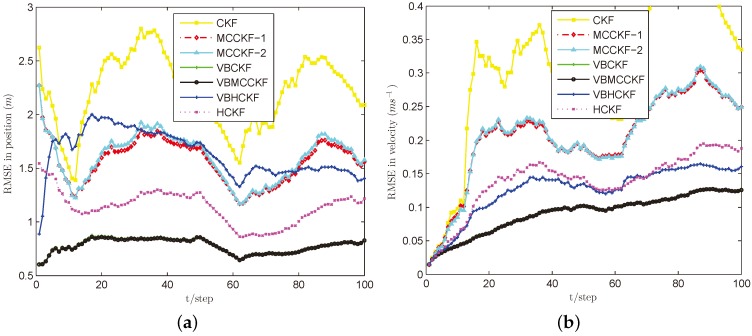
RMSE performances of different filters under Case C. (**a**) Position; (**b**) Velocity.

**Figure 5:**
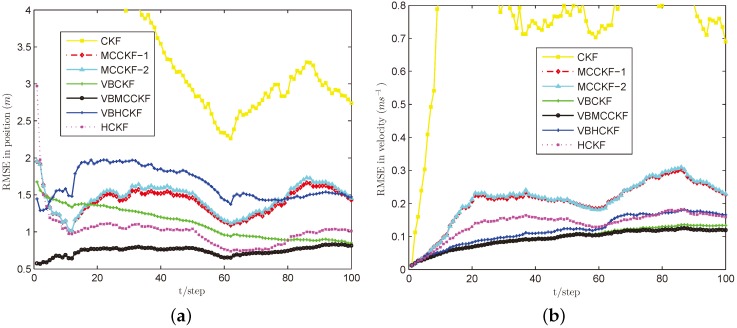
RMSE performances of different filters under Case D. (**a**) position; (**b**) velocity.

**Figure 6:**
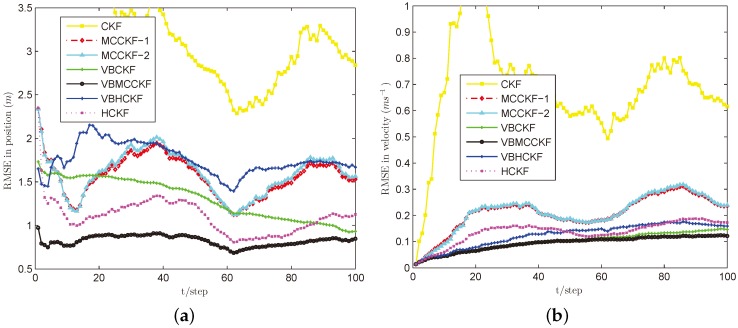
RMSE performances of different filters under Case E. (**a**) position; (**b**) velocity.

In these simulations, we use σ=8 for MCC based algorithms, the commonly used threshold h=1.345 for Huber’s function, and we set ρ=0.8 and N=3 for VB approximations. We perform L=100 steps and run M=100 Monte Carlo experiments for each case. The simulation results are plotted in Figure 2, Figure 3, Figure 4, Figure 5 and Figure 6.

Under the Gaussian measurement noise with known noise covariance, as given in Figure 2, both MCCKF and HCKF have nearly similar estimation accuracy to CKF, since they will reduce to CKF if choosing proper free parameters (e.g., the σ and *h* are infinity). VBCKF and VBMCCKF work slightly worse as compared with CKF because they only use their online estimated measurement noise covariance instead of the real one. In particular, the VBHCKF has the worst performance since the commonly used parameter h=1.345 for Huber’s function doesn’t fit the Gaussian noise situation when using the inaccurate online estimated measurement noise covariance. The proposed VBMCCKF works well with the same kernel bandwidth under both Gaussian and non-Gaussian noise situations, as will be shown in the following cases.

Figure 3, Figure 4, Figure 5 and Figure 6 show the estimation performances of different algorithms under Cases B–E. It can be seen obviously that CKF has the worst estimation accuracy since it requires the measurement noise satisfying Gaussian distribution with known covariance, which is violated in these situations. MCCKF-1 and MCCKF-2 have similar estimation performance but are slightly worse than HCKF when using this kernel bandwidth. As demonstrated in [23,24,25,26,27], MCCKF is able to obtain better estimation accuracy than HCKF with a suitable σ. The estimation results of MCCKF and HCKF do not change too much when Gaussian mixture noise or shot noise are added, since they are robust filters. The VBHCKF has better estimation results in velocity but worse accuracy than HCKF. The VBCKF has much better estimation in Cases B and C, as it is able to online estimate the time varying measurement noise covariance. However, its performance will degrade once shot noise is injected in Cases D and E. Among these algorithms, our VBMCCKF has the best estimation accuracy as compared with other algorithms under Cases B–E. It shows the adaptivity to unknown changing measurement noise covariance and robustness to Gaussian mixture noise and shot noise. Its estimation results are also much better than VBHCKF since the MCC has the potential to capture high order information than Huber’s function. The ARMSEs of these filters under different noises are also given in Table 1 to clearly show the differences.

### 4.2. Field Results of Integrated Navigation

To further illustrate the effectiveness of the proposed algorithm, we compare our algorithm and existing related methods using the real data collected by a self-made fiber optical gyroscope inertial navigation system (INS) together with a doppler velocity logger (DVL). The integrated navigation results of photonics inertial navigation system (PHINS) and GPS are used as the reference system. We adopt the loosely coupled method to fusion the information of INS and DVL. The state vector is chosen as $x={\left[\delta L\;\delta \lambda \;\delta V_{E}\;\delta V_{N}\;\varphi_{x}\;\varphi_{y}\;\varphi_{z}\;\nabla_{x}\;\nabla_{y}\;\nabla_{z}\;\varepsilon_{x}\;\varepsilon_{y}\;\varepsilon_{z}\right]}^{T}$, where δL and δλ are the latitude and longitude error, $\left\{\delta V_{j},\varphi_{j},\nabla_{j},\varepsilon_{j}\right\}$ are the velocity error, attitude error, accelerometer bias and gyroscope constant drift, respectively. *j* denotes the subscribe {e,n,x,y,z}, where *e* and *n* present the east and north directions in the local-level frame, and *x*, *y*, and *z* are the directions of three axises in the body frame. Then, the continuous system model is given as follows:

(64) $$\dot{x}\left(t\right)=A\left(t\right)x\left(t\right)+B\left(t\right)w\left(t\right),$$

where *t* is the continuous system time, and $w\left(t\right)={\left[0_{1\times 2}\;w_{ax}\;w_{ay}\;w_{gx}\;w_{gy}\;w_{gz}\;0_{1\times 5}\right]}^{T}$ is the process noise, which contains the Gaussian noise of both accelerometers and gyroscopes. The detailed elements of matrix A(t) and B(t) can refer to [34]. The measurement equation is

(65) z(t)=Hx(t)+v(t),

where $z\left(t\right)={\left[\begin{array}{cc} \left(V_{e}^{INS}-V_{e}^{DVL}\right) & \left(V_{n}^{INS}-V_{n}^{DVL}\right) \end{array}\right]}^{T}$, $H=[0_{2\times 2}\;I_{2\times 2}\;0_{2\times 8}]$, and $v\left(t\right)={\left[V_{e}^{DVL}\;V_{n}^{DVL}\right]}^{T}$. Then, the discretization process is performed before running filtering algorithms.

We compare the estimation results of KF, VBKF, HKF, MCKF, and VBMCKF, where we choose h=1.345, σ=4, ρ=0.96, and N=3. We set ${\hat{x}}_{0}={\left[0_{1\times 12}\right]}^{T}$, and the covariance is ***P***_0_ = diag($\begin{array}{cc} \frac{500}{Re}\;\mathrm{rad}/s, & \frac{500}{Re}\;\mathrm{rad}/s, \end{array}$ 0.5°, 0.5°, 3°, 1 × 10^−4^ *g*, 1 × 10^−4^ *g*, 0.01°/h, 0.01°/h, 0.01°/h), where Re is the radius of the earth, and *g* is the gravitational acceleration. The process noise covariance and measurement noise covariance are set as $Q_{i}$ = diag(0, 0, 1 × 10^−3^ *g*, 1 × 10^−3^ *g*, 0.025°/h, 0.025 °/h, 0.025 °/h, 0, 0, 0, 0, 0) and $R_{i}$ = diag(0.1 m/s, 0.1 m/s) according to the parameters of INS and DVL.

**Figure 7:**
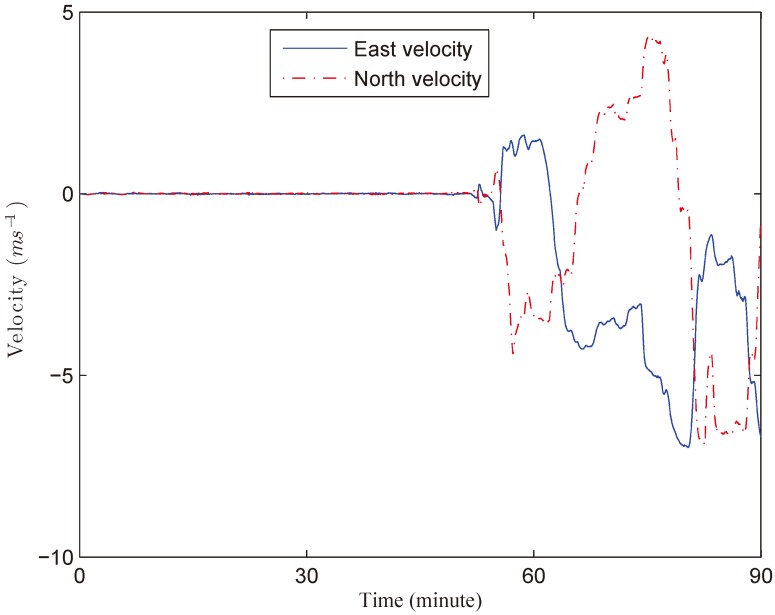
Real velocities of this field experiment.

**Figure 8:**
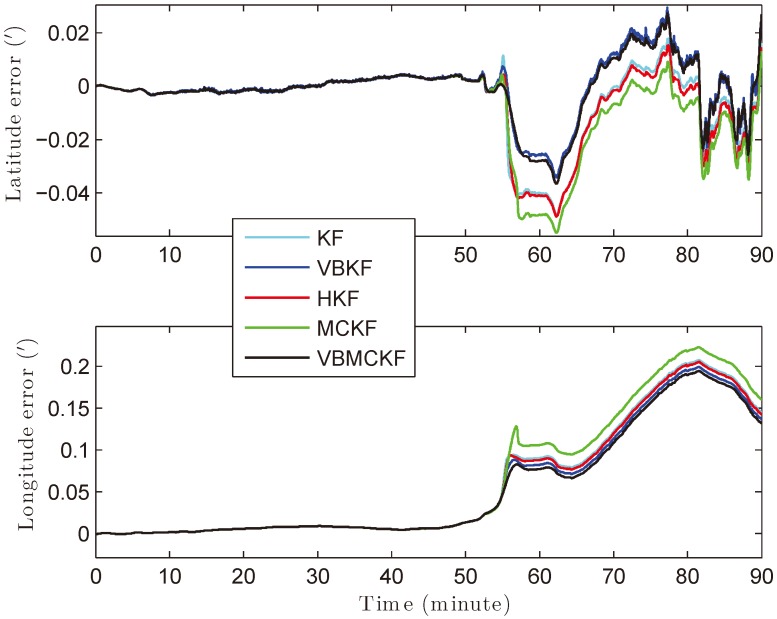
Position errors of different filters.

**Figure 9:**
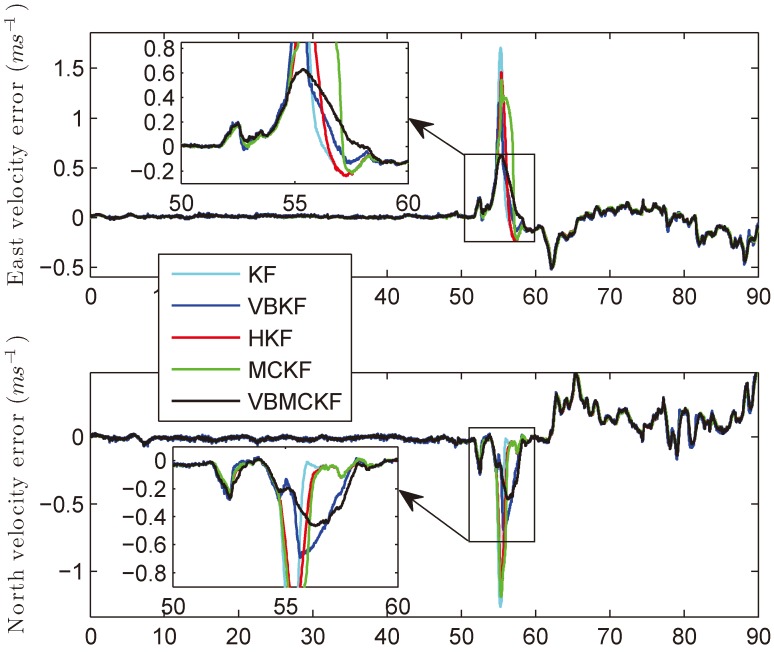
Velocity errors of different filters.

In this experiment, the system is first tested in anchorage for about 50 min, then the ship starts to move. The real velocities of the ship are shown in Figure 7 provided by the commercial INS/GPS integrated navigation system. The collected data is processed using MATLAB (R2014a by MathWorks, Inc., Natick, MA, USA) on a computer with 2.50 GHz Intel Core i5-7300HQ CPU and 8 GB memory. The total computational time of KF, VBKF, HKF, MCKF, and VBMCKF are 0.1900 s, 0.4800 s, 0.2640 s, 0.2310 s, and 0.6340 s, respectively. The position and velocity errors of different filters are given in Figure 8 and Figure 9. The differences between attitude and heading errors are quite similar so we omit them.

It can be seen from Figure 8 and Figure 9 that when the motion state changes sharply, the proposed VBMCKF algorithm has the smallest estimation errors with slightly increased computational time as compared with other estimation methods.

## 5. Conclusions

In this paper, a novel adaptive MCGF based on VB approximation is proposed. The MCC is used to reduce the effect of non-Gaussian measurement noise and outliers, while we use VB to estimate the unknown measurement noise covariance. Experimental results based on simulation examples and real data show that the proposed algorithm has better estimation accuracy than related robust and adaptive filters.

## Figures and Tables

**Table 1:** ARMSEs of different filters under five cases.

Algorithms	Case A		Case B		Case C		Case D		Case E	
	Pos.	Vel.	Pos.	Vel.	Pos.	Vel.	Pos.	Vel.	Pos.	Vel.
CKF	0.4097	0.0855	2.2930	0.3121	2.2050	0.3053	3.7720	0.7809	3.2960	0.6923
MCCKF-1	0.4077	0.0854	1.4960	0.1959	1.5680	0.2052	1.4090	0.2077	1.5680	0.2100
MCCKF-2	0.4079	0.0853	1.5280	0.1989	1.5990	0.2058	1.4500	0.2098	1.6010	0.2114
HCKF	0.4045	0.0847	1.0990	0.1304	1.1360	0.1390	1.0100	0.1384	1.1030	0.1365
VBHCKF	0.6500	0.1296	1.7180	0.1185	1.6360	0.1260	1.6460	0.1207	1.7590	0.1251
VBCKF	0.4362	0.0880	0.8761	0.0845	0.7828	0.0914	1.1240	0.0977	1.3100	0.1000
VBMCCKF	0.4350	0.0878	0.8633	0.0843	0.7752	0.0909	0.7424	0.0933	0.8236	0.0931
